# The Transactions of the Medical Association of Georgia

**Published:** 1885-05-20

**Authors:** 


### EDITORIALS AND MISCELLANEOUS.

### THE TRANSACTIONS OF THE MEDICAL ASSOCIATION OF GEORGIA.

It will be seen by reference to the abstract of the late proceedings of the Association at Savannah that the Transactions are hereafter to be published in the Atlanta Medical Journal of this city. The only ground which appears to be offered for this action is the "*lack of funds*," which means that the members declined to pay their dues, which amounted to the sum of $3.00 per annum for each member. Of this, enough could not be collected to publish the Transactions in separate form; and yet the Secretary, who runs a medical Journal, and who is also Chairman of the Publication Committee, proposes to publish the Transactions in his Journal at $2.00 per member, each member to receive his Journal by paying him $2.00. This, at first sight, appears to be one-third less than it has heretofore cost; but, as there are 268 members, it really binds the society to the extent of $2.00 for each member, or the sum of $536.00, an amount exceeding that which was expended on the last volume of the Transactions.

Complaint is made of the nonpayment of dues and the necessity of procuring advertisements to supplement the receipts. The question as to *the cause*

*of the great indifference of the members* in the matter of paying their dues seems not to have been thought of. Nor did they consider the impropriety and unfairness of giving the influence and strength of the Association to one journal when there was another prominent journal in the State, the friends of which would have cause to be offended, thus jeopardizing the harmony of the Profession in the State.

We will add that it is regarded by many of the true friends of the Profession throughout the State as unfortunate and unwise, as it is likely to spring a question as to the underlying motives that prompted it, and lead to trouble in our ranks.

The editors of this Journal, it is well known, have always been true to the Profession, to the ethics and the principles of right and justice. It is true we have not scrupled to speak the truth, or to condemn the wrong when it was necessary; and, though we have a large circulation and numerous friends throughout the State and the South, we never would have presumed to ask such a thing of the Association as has been done in this instance, feeling, as we do, that such action would have placed us and the Society in an unfavorable attitude, and jeopardized the best interests of our State Association.

Thus much we have felt it a duty to say as Journalists. As defenders of the "honor and good of the Profession," we are willing to discuss, in an unprejudiced and fair manner, the action referred to, and we distinctly aver that, in what we have said, we do not impugn the Association at large or the members who innocently supported this action: but it is for the defense of the members and the honor of the Profession that we have spoken. We feel assured our impressions on this matter are correct, and that our views will be sustained by unprejudiced minds in the Profession everywhere.

